# Transcranial alternating current stimulation (tACS)

**DOI:** 10.3389/fnhum.2013.00317

**Published:** 2013-06-28

**Authors:** Andrea Antal, Walter Paulus

**Affiliations:** Department of Clinical Neurophysiology, Georg-August University of GöttingenGöttingen, Germany

**Keywords:** tACS, oscillations, human brain, motor, visual

## Abstract

Transcranial alternating current stimulation (tACS) seems likely to open a new era of the field of noninvasive electrical stimulation of the human brain by directly interfering with cortical rhythms. It is expected to synchronize (by one single resonance frequency) or desynchronize (e.g., by the application of several frequencies) cortical oscillations. If applied long enough it may cause neuroplastic effects. In the theta range it may improve cognition when applied in phase. Alpha rhythms could improve motor performance, whereas beta intrusion may deteriorate them. TACS with both alpha and beta frequencies has a high likelihood to induce retinal phosphenes. Gamma intrusion can possibly interfere with attention. Stimulation in the “ripple” range induces intensity dependent inhibition or excitation in the motor cortex (M1) most likely by entrainment of neuronal networks, whereas stimulation in the low kHz range induces excitation by neuronal membrane interference. TACS in the 200 kHz range may have a potential in oncology.

## Introduction

Transcranial alternating current stimulation (tACS)—the external application of oscillating electrical currents—is able to influence cortical excitability and activity (Antal et al., 2008; Chaieb et al., 2011; Moliadze et al., 2012; Wach et al., 2013). With exceptions (Marshall et al., 2006; Neuling et al., 2012) tACS is applied in most studies without a DC offset. It's simple form uses sinusoidal stimulation; however, any other waveform appears possible, such as rectangular current shapes. Ten Hz tACS was modeled in a realistic head model and was suggested to generate larger and more focused fields than DC stimulation (Manoli et al., 2012); with applied frequencies of 100 up to 1000 Hz a decrease of the size of this electrical field was assumed. This claim was, however, only based on differences in skin resistance at low frequencies which renders the statement quite doubtful (Paulus and Opitz, 2013). The major parameters that can shape the direction and the duration of the tACS-induced effects are the frequency, the intensity and the phase of the stimulation. The effect of duration of tACS on motor evoked potential (MEP) has not been systematically investigated yet. Increasing the duration of transcranial direct current stimulation (tDCS) results in a prolongation of the induced aftereffects (Nitsche and Paulus, 2000) up to about 13 min whereas doubling the 13 stimulation to 26 min inverses MEP aftereffects into inhibition (Batsikadze et al., 2013) It is unclear if this can be translated to tACS, too.

## ACS effects in the normal brain

### Frequency of the stimulation

tACS may be applied in a wide frequency range. At present data are available between close to DC up to 5 kHz for plasticity studies (Chaieb et al., 2011) and 200 kHz for tumor therapy (Kirson et al., 2007) using a single frequency. However, any combination of frequencies is possible: one special form of tACS is transcranial random noise stimulation (tRNS), which so far has been studied with a frequency spectrum between 0.1 Hz and 640 Hz with a “white noise” characteristic (Terney et al., 2008).

tACS applied at conventional EEG frequencies (0.1–80 Hz) and in the so called “ripple” range (140 Hz, see below) (Moliadze et al., 2010) may be able to interact with ongoing rhythms in the cortex. A very low frequency (0.75 Hz) stimulation combined with DC offset during non-rapid-eye-movement sleep in healthy humans enhances the retention of hippocampus-dependent declarative memories when tested the next morning (Marshall et al., 2006). The DC offset used in this study leaves open the possibility of a DC effect (Bergmann et al., 2009).

Effects of tACS applied in the EEG range might differ depending on the outread parameters. A trend toward MEP inhibition following 10 Hz AC stimulation over the primary motor cortex (M1) was observed (Antal et al., 2008), while 10 Hz stimulation improved visuomotor implicit learning, using a serial reaction time task. In the MEP measurement shorter stimulation duration (5 min) was applied whereas tACS in the implicit learning study lasted about twice as long. Nevertheless, a dissociation between MEP excitability changes and implicit learning under tACS has already been described (Moliadze et al., 2010). 140 Hz stimulation induces the largest MEP increase, whereas only 250 Hz improved implicit motor learning.

In another study, whereas 20 Hz tACS over the M1 increased corticospinal excitability (Feurra et al., 2011) as measured by MEP size, it slowed down voluntary movements using a visuomotor task (Pogosyan et al., 2009) but in parallel it increased beta coherence between scalp-recorded activity and electromyographic activity (EMG) of the first dorsal interosseus muscle. Opposing effects at beta and gamma frequencies depending on phase of a of motor task exist (Joundi et al., 2012): using a visually driven go—no-go task stimulation at 20 Hz afforded a significant but modest slowing of force production in the go task, however, stimulation in no-go trials, where the triggered motor task involved inhibition, led to a major reduction in force generation. In contrast, 70 Hz tACS was ineffective during errors of commission following no-go cues, but increased performance during go trials.

TACS up to 80 Hz elicits phosphenes in a frequency- and intensity dependent way (Turi et al., 2013). When applied over the occipital cortex, the perception of phosphenes was peaking at about 15 Hz in brightness with a lower peak in darkness (Kanai et al., 2008). Although electrodes were placed over Oz and Cz, this effect was probably induced by far field stimulation at the retina (Schutter and Hortensius, 2010). TACS can probably only influence visual cortical functions at a subthreshold level as shown by modification of transcranial magnetic stimulation (TMS)-induced phosphene-thresholds (Kanai et al., 2010). Furthermore, contrast-discrimination thresholds were decreased only during 60 Hz tACS, but not during 40 and 80 Hz stimulations (Laczo et al., 2012).

TACS applied over the PO9 and PO10 EEG electrode positions at the individual alpha frequency range (8–12 Hz) induced an entrainment of the applied oscillatory activity (Zaehle et al., 2010). However, it was recently documented that the after-effects of tACS applied at the individual alpha frequency may depend on the individual endogenous power: tACS was effective only under conditions of low endogenous alpha frequency power (Neuling et al., 2013). Furthermore, when stimulation frequency was fixed at 6 and 10 Hz, tACS impaired performance in the visual detection task (Brignani et al., 2013).

tACS applied outside the conventional EEG frequency range, e.g., with frequencies of 140 Hz and in the low kHz range (1–5 kHz) increases excitability in a similar way than anodal tDCS, when 1 mA intensity is used (Moliadze et al., 2010; Chaieb et al., 2011). Stimulation at 80 Hz remains without an effect, while 250 Hz clearly had a delayed onset and shorter lasting response, compared to the MEP increase observed during and after 140 Hz tACS.

The tRNS paradigm was developed with a potential to desynchronize normal and pathological cortical rhythms (Terney et al., 2008). The rationale behind this method is a possible entrainment with cortical oscillations of different frequencies at the same time. This may apply for intra-areal with higher oscillation frequencies or for inter-areal oscillations with lower frequencies. Input noise plays a role in sensitizing neuronal systems through a mechanism known as stochastic resonance (Wiesenfeld and Moss, 1995). Alternatively, impaired signal detection might be improved by input noise in order to sensitise sensory processing (Moss et al., 2004). An excitability increase lasting up to 90 min, observed both for MEP measures and behavioral tasks, was induced after 10 min of tRNS. Unexpectedly higher frequencies (100–640 Hz) and not frequencies less than 100 Hz were responsible for this excitability increase.

The efficacy of the stimulation seems to be dependent on the type of the task and on the power of intrinsic oscillations at baseline (Neuling et al., 2013) and the involvement of the different memory systems in a given cognitive task: when tRNS was applied over the dorsolateral prefontal cortex (DLPFC) subjects made more mistakes in a probabilistic classification task (Ambrus et al., 2011), whereas when using the n-back task no significant change in performance was found (Mulquiney et al., 2011). When tRNS was applied of the visual cortex improved neuroplasticity in a perceptual learning paradigm (Fertonani et al., 2011). Nevertheless, the neuronal mechanisms underlying the effect of tRNS might be different from those of tACS, using a single stimulation frequency.

### Intensity of the stimulation

The effect of tACS appears to be intensity dependent. A trend in a first study (Antal et al., 2008) using a low intensity of 0.4 mA over the M1 toward MEP inhibition following 10 Hz AC stimulation was confirmed later with higher frequencies (Moliadze et al., 2012). Other tACS frequencies between 5 and 40 Hz failed to induce any measurable aftereffects at this (too) low intensity.

Interestingly, both tACS at 140 Hz and tRNS show an intensity-dependent aftereffect: whereas 0.2 mA intensity has no effect an intensity of 0.4 mA leads to inhibition, 0.6 and 0.8 mA do not provide a significant effect (Moliadze et al., 2012). With 1 mA an increase of the MEP amplitudes can be seen. This suggests that inhibitory circuits can be excited preferentially with lower intensities, an effect which has also been documented for TMS (Berger et al., 2011). Nevertheless, the reason for this observed reversal in the direction of MEP effects induced by higher frequency stimulation at different intensities has not been clarified yet. It is likely that 140 Hz and tRNS at the lower intensity only facilitate intracortical inhibitory networks of corticospinal motoneurons, thus resulting in net inhibition of MEP amplitudes (Pashut et al., 2011). It also cannot be excluded that stimulation applied at 0.4 mA may inhibit intracortical facilitatory effects on corticospinal motoneurons. When recording in a pyramidal neuron located in layer 5 of the rat cortex the composite response to an electrical stimulation of various layers (2–3, 4, or 6), in terms of excitation–inhibition balance, resulted in conductance changes consisting of 20% excitation and 80% inhibition, independent from the stimulated layer. Moreover, it was shown that excitatory circuits are strongly controlled by inhibitory circuits (Maffei et al., 2004) by feedback and feed-forward connections (Bannister, 2005).

### Phase of the stimulation

Brain oscillations are characterized in addition to frequency and power by their phase. Modeling studies propose that in active neuronal networks weak electrical fields can induce small but coherent changes in the firing rate and timing of neuronal populations that can be magnified by dynamic network activity (Radman et al., 2007; Reato et al., 2010) When stimulating the left frontal and parietal cortex by 6 Hz tACS in phase, cognitive performance in a delayed letter discrimination task was improved, when stimulating out of phase it was delayed (Polania et al., 2012). In a recent study stimulating the temporal cortex using 10 Hz with DC-offset it was found that manipulation of the phase resulted in different auditory detection thresholds, which supports the notion that perception can be periodically modulated by oscillatory processes (Neuling et al., 2012). Nevertheless, the DC offset used in this study leaves open the possibility of a DC effect.

## Mechanisms of action

tACS applied in the EEG range is believed to mainly entrain with or synchronize neuronal networks, thus inducing changes in ongoing oscillatory brain activity. Indeed, spike synchrony of converging input has been shown to enhance the information transfer and speed up processing (e.g., Butts et al., 2007). Nevertheless, stimulation applied in the kHz range probably does not interfere with oscillatory activity, but targets the membrane excitability of neurons more selectively. It could be that the temporary modification of the synapse once exposed to a rapidly alternating electrical field, alters the associated biochemical mechanisms, such as accumulation of calcium in the presynaptic nerve terminals leading to short-term synaptic plasticity effects (Citri and Malenka, 2008).

The mechanisms of tRNS so far are unclear, if e.g., repeated opening of Na^+^ channels or a higher sensitivity of neuronal networks to electrical field modulation than the single neuron threshold (Francis et al., 2003).

## Applications in disease

tACS would have a particular indication in disorders in which abnormal oscillatory patterns may play a role, such as Parkinson's disease or schizophrenia (Gonzalez-Burgos and Lewis, 2008; Burns et al., 2011) by attenuating or resetting anomalous oscillations. Indeed, Parkinsonian resting tremor could be bisected by tACS of the M1 at specified phase alignments (Brittain et al., 2013).

Using 200 kHz frequency a pilot clinical trial was carried out treating human patients suffering from recurrent gliobastoma (Kirson et al., 2007). By transcranial application of continuous high frequency stimulation inhibits the growth of this treatment-resistant tumor, with little or no side effects, pursuing the concept that dividing tumor cells can be destroyed during mitosis.

Applying the current transorbitally at the individual phosphene thresholds ACS is effective in the therapy following optic nerve injury in human (Gall et al., 2010; Sabel et al., 2011).

## Comparison to rTMS?

Both rTMS and tACS could provide the basis to interact with or induce local, probably also remote oscillatory activity. While rTMS involves delivering a brief, repetitive, high-intensity magnetic pulses to the head through a coil that induces electrical currents in a focal area underneath this area, with regard to tACS, oscillatory current is delivered with a battery-driven stimulator by means of a large electrode located on the area of interest and a reference electrode that is placed over a neutral area. tACS has some advantages compared to rTMS: (1) it is clearly cheaper due to the small and compact equipment; (2) it can be more easily combined with online cognitive projects; (3) it produces no acoustic noise and muscle twitching of cranial muscles and it causes much less or no perceptual skin sensations (Ambrus et al., 2010; Turi et al., 2013) and is hereby more suitable for double-blind, sham-controlled studies. Nevertheless, there are disadvantages, including shunting of the electric currents through the scalp and the skin irritations that sometimes can be observed under electrodes.

## Future directions

tACS is only in its beginnings. A seemly indefinitely number of stimulation paradigms will have to be condensed to those with highest physiological relevance. As a prerequisite, this requires a clearer picture of the neuronal mechanisms involved in tACS-induced entrainment. Knowledge of their dynamics over time would enable to formulate optimized protocols for future tACS studies.

### Conflict of interest statement

The authors declare that the research was conducted in the absence of any commercial or financial relationships that could be construed as a potential conflict of interest.
